# A Systematic Literature Review of LoRaWAN: Sensors and Applications

**DOI:** 10.3390/s23208440

**Published:** 2023-10-13

**Authors:** Vicky Bonilla, Brandon Campoverde, Sang Guun Yoo

**Affiliations:** 1 Departamento de Informática y Ciencias de la Computación, Escuela Politécnica Nacional, Quito 170525, Ecuador; 2 Smart Lab, Escuela Politécnica Nacional, Quito 170525, Ecuador

**Keywords:** LoRaWAN, sensors, applications, LoRaWan devices

## Abstract

LoRaWAN is a communication protocol designed especially for Internet of Things (IoT) applications that offers benefits such as long-distance connection and low power consumption. Due to the characteristics of LoRaWAN, this technology has gained great popularity in various IoT applications, such as environmental monitoring, smart agriculture, and applications in the areas of health and mobility, among others. Given this situation, the objective of this work is to provide an in-depth overview of LoRaWAN technology in terms of its applications, as well as the devices that have been used for the development of such applications. Additionally, this work reviews what other areas of LoRaWAN have been covered in different scientific articles, i.e., performance improvement and security. Among the main results of this study though analyzing previous works, we can say that most of them have been developed in the area of environmental monitoring and have used low-cost devices such as Arduinos, Raspberry Pis, and relatively low-cost commercial products such as those of the Semtech and STMicroelectronics brands. The analysis of the present work shows objectively and formally that LoRaWAN technology can be applied in various applications and that there are many studies that try to optimize its performance and security. This paper seeks to identify and describe the most relevant applications of LoRaWAN in different sectors, such as agriculture, health, and environmental monitoring, among others, and the challenges and solutions found in each area. This literature review will provide a valuable reference to understand the potential and opportunities offered by LoRaWAN technology.

## 1. Introduction

Internet of Things (IoT) refers to the interconnection of electronic devices that have the ability to collect and transmit data over the Internet. IoT allows the automation of processes and thus achieves greater efficiency when making decisions through the collection of data in real time. Both IoT technology and its applications have grown tremendously in recent years and have been used in different fields, ranging from smart homes to smart city management and mass transportation [1].

Currently, there are a large number of technologies that provide communication to IoT systems. The selection of one of these technologies depends on certain criteria such as coverage area, data transmission rate, and power consumption. Among the communication options for IoT systems, low-power wide-area networks (LPWAN) stand out, which are characterized by their wide coverage, low energy consumption, and high scalability [2]. Among the different LPWAN technologies, the most popular are Sigfox, LoRaWAN, and NB-IoT.

Sigfox is a proprietary low-power-consumption communication technology that operates on frequencies of 868 MHz in Europe and 902 MHz in United States. This technology uses a license-free frequency band to allow data transmission over long distances. Sigfox focuses on energy efficiency and offers wide global coverage but with a slower data rate. Sigfox’s network architecture allows messages from one end device to be received by multiple base stations [3].

Narrow-band IoT (NB-IoT), also called LTE Cat NB1, is a 3GPP-standard-based narrow-band radio technology that was published in 2016. It is a low-speed and low-power network that offers higher security and data transmission speed in comparison with Sigfox. However, NB-IoT requires existing cellular infrastructure and can be more expensive to implement [4].

LoRaWAN, or long-range wide-area network, is an LPWAN network technology that uses spread spectrum technology to enable data transmission over long distances. LoRaWAN offers high energy efficiency and wide coverage, as well as the ability to send data through obstacles such as buildings and walls. Furthermore, LoRaWAN is an open network, which means that it is not controlled by a single provider and can be used by multiple companies and organizations [5].

Among the aforementioned options, LoRaWAN is an attractive choice due to its high energy efficiency, wide coverage, and ability to send data through obstacles. In addition, since LoRaWAN is an open and flexible network, it can be used by companies and organizations without any kind of restrictions, which makes it the most accessible and affordable option for many applications. LoRaWAN is primarily designed for being used with sensors, which are expected to exchange information with a server at a low transmission rate and relatively long intervals of time where a transmission can take place every hour or even days apart [6]. LoRaWAN is used globally, and its use is constantly expanding, which makes it a very attractive solution for future IoT-based applications [6].

LoRaWAN operates with tree main components: end devices, gateways, and back-end servers (a back-end server has a network server (NS), join server (JS), and application server (AS)) [7].

LoRaWAN has three classes of end devices:

- Class A: Bidirectional devices with the highest latency among the three classes. These devices are in sleep mode until they receive an uplink message which opens two reception windows and enables a downlink [4].
- Class B: Like class A, these are bidirectional devices. These devices open additional reception windows at scheduled times in addition to the two default windows [4].
- Class C: The reception windows of this class keep open continuously; due to this, these devices consume the greatest amount of energy of the three classes [4].

LoRaWAN incorporates robust security mechanisms to protect data integrity and confidentiality. For encryption purposes, LoRaWAN uses AES-128 in CTR mode, whereas a message authentication code (MAC) is used for integrity [7].

This technology supports two activation methods: activation by personalization (ABP) and over-the-air activation (OTAA). ABP is a straightforward method where device parameters, such as DevEUI, AppEUI, and AppKey, are pre-configured in the end node, AS, NS, and JS for joining and sending messages. This could be insecure since the session keys are always the same. On the other hand, OTAA is a more robust and secure activation method. The end devices in OTAA mode start a join procedure with DeviceEUI, Join EUI, NwkKey, and AppKey. With these parameters, dynamic session keys are generated and exchanged every session [7].

With this background, the present work seeks to provide a complete and updated overview of the areas in which LoRaWAN long-range communication technology has been used in the last 5 years. A formal literature study has been carried out through the analysis of scientific articles indexed in international scientific databases such as IEEE Xplore, Springer Link, SCOPUS, and ACM Digital Library. This paper seeks to identify and describe the most relevant applications of LoRaWAN in different sectors, such as agriculture, health, and environmental monitoring, among others. In addition, the challenges and solutions found in each area by previous works will be analyzed, as well as their used technologies. This literature review will provide a valuable reference to understand the potential and opportunities offered by LoRaWAN technology, as well as to identify its trends. Additionally, this work will help to identify the current limitations and how to improve them in the future.

The following article aims to answer several questions which will help the reader understand the state of the art of LoRaWAN, i.e., What are the primary applications within the realm of LoRaWAN? Which brands of boards and modules are most employed? What advances are currently underway in the field of LoRaWAN technology?

## 2. Materials and Methods

The development of the systematic review of the literature was carried out in the area of LoRaWAN applications. For this, the methodology proposed by [8] was used. This methodology is composed of several phases, i.e., planning, automatic review of results, and manual review of results. 

### 2.1. Planning

In the planning phase, the keywords and databases on which the search would be carried out were defined. For the search, the following keywords were used: “LoRaWAN”, “sensors”, and “applications”, since the intention of this work is to analyze the different applications developed using LoRaWAN technology. Once the keywords were defined, these terms were searched in the following scientific databases: IEEE Xplore, Springer Link, SCOPUS, and ACM Digital Library. These databases were selected because they contain scientific information focused on the area of computer science.

### 2.2. Automatic Review of Results

In this phase, the search strings defined in the planning phase were applied in the established databases. The search results are shown in Table 1. The table contains the name of the digital repository, the search string, and the number of articles found.

As a result of the search, 2588 documents were found. To reduce the number of articles to those of interest for the present study, a more specific search string was generated, which is shown in Table 2. In this second search, the number of articles was limited to those including the search keyworks in their abstract. Additionally, the year of publication of the articles was limited to the last 5 years since it is the common period taken for technological surveillance.

From the results obtained in the second search, it was decided to carry out a third search so that the set of study works were more exact. For the third search, exact keywords such as “Internet of Things” and “LoRaWAN” were also included. The search strings can be seen in Table 3.

As a last filter, to reduce the number of articles in the results of the SCOPUS database, the word “monitoring” was included. In this way, the results of Table 4 were obtained.

### 2.3. Manual Review of Results

Finally, from the search results of the fourth search, we proceeded to read the abstracts and manually choose the most relevant works for the research objectives. Likewise, Mendeley was manually searched for important related articles, from which 2 more articles were obtained. From this process, the results of Table 5 were obtained. 

## 3. Results

Analyzing the applications of LoRaWAN and the devices used in those applications is important for several reasons:

- Knowledge Acquisition: First of all, conducting a literature review allows new researchers to gather the existing knowledge and insights about LoRaWAN applications. It can help to understand the current state of research, identify trends, and gain a comprehensive understanding of the subject matter.
- Identifying Gaps and Research Opportunities: A literature review enables the identification of gaps or areas that have not been extensively studied; by analyzing the existing scientific papers, it is possible to identify unanswered research questions or areas where further research is required.
- Validation and Evaluation: Scientific papers provide valuable insights and data on the applications of LoRaWAN and the devices used. By reviewing and evaluating these papers, you can assess the methodologies, experimental setups, and results presented. This evaluation helps you validate the findings and conclusions, ensuring that your work is based on solid scientific evidence and contributes to the overall reliability of the field.
- Keeping Up with Emerging Trends: The field of LoRaWAN applications is continuously evolving. By reviewing scientific papers, you can stay up to date with the latest advancements, emerging trends, and innovative approaches. This knowledge of current research allows you to incorporate the latest findings into your work and ensures that you are aware of the state of the art in the field.

For the aforementioned reasons, the present work presents a literature review of the scientific papers on the applications of LoRaWAN and the devices used for helping new researchers acquiring knowledge in the area, identifying research gaps, validating findings, and staying informed about emerging trends.

### 3.1. Applications of LoRaWAN

LoRaWAN finds applications in diverse fields such as smart cities, industrial monitoring, agriculture, smart buildings, asset tracking, and environmental monitoring. In smart cities, it enables the efficient management of resources like lighting, waste, parking, and environmental conditions. In industrial settings, LoRaWAN enables remote monitoring of equipment, predictive maintenance, and supply-chain optimization. In agriculture, it facilitates soil and irrigation monitoring, crop management, and livestock tracking. In smart buildings, LoRaWAN is utilized for energy management, security systems, and occupancy sensing. Asset tracking across industries and environmental monitoring of air and water quality are additional areas where LoRaWAN plays a crucial role. To be more concrete, Table 6 classifies the different previous works analyzed in this review.

From Table 6, it can be seen that most of the previous works focus on the area of environmental monitoring, followed by works referring to other areas such as performance improvement in LoRaWAN technologies. Works about environmental monitoring applications are the most popular, perhaps due to the diversity of sensors on the market for the measurement of different parameters such as UV radiation, temperature, humidity, and CO_2_, among others. The works belonging to “Other areas” are comparative studies of the general applications and performance of LoRaWAN technology. 

In the following subsections, we will describe the different works developed in the different areas of application.

#### 3.1.1. Agriculture

Agriculture is a sector of special importance due to the food demands of the growing population. In this context, different works have been developed to monitor various aspects that are required for agriculture, ranging from soil quality to crop monitoring. In [9], a remote crop monitoring system was developed using cost-effective sensors that measure parameters such as pH, temperature, and humidity; in this work, the gathered data are sent to a The Things Network (TTN) server. In [10], a prototype based on LoRaWAN was proposed that allows measuring the humidity, temperature, and electrical conductivity of the soil.

On the other hand, in [13], the importance of security in the field of agriculture was emphasized by implementing a hardware secure module (HSM). This module made it possible to encrypt the secret keys and store the encrypted keys on a Raspberry Pi. The decryption of these keys is only possible if there is access to the HSM.

In [11], the use of wireless underground sensor networks (WUSNs) was proposed, which provide coverage of a 7 km radius with a depth of up to 50 cm. The results indicate that WUSNs are not scalable if message-delivery-confirmation packets are required. When the nodes were buried 50 cm, the coverage distance was up to 160 m. Additionally, in [12], an air-quality monitoring system was implemented. In this work, nine gateways were placed in six different locations; it was shown that a packet reception could be obtained from 72.4% of the messages sent. The implementation of 100 air monitoring devices and four additional gateways is proposed to test the scalability of this system.

#### 3.1.2. Environmental Monitoring

Environmental monitoring is the most frequent application of LoRaWAN among the previous works analyzed in this paper. Among these applications, it can be seen that the measurement of ultraviolet (UV) rays, the monitoring of air quality, and the monitoring of water stand out.

In [14], a monitoring system was created to assist in the prevention of diseases caused by solar radiation through Twitter alerts using LoRaWAN technology and ThingSpeak software. In [18], the UV-radiation measurement procedure was also performed using an ESP32 controller. In [19], commercial sensors were used to implement the measurement of ultraviolet radiation with an optimization of energy that allows them to work up to 14 days; communication was also optimized for distances greater than 0.7 km. In [24], the design of a device based on UV optical sensors that allows determining the existing radiation index was presented; ultraviolet radiation index (UVI) levels were displayed through a graphical user interface in real time, which also allowed generating a report in a .csv file. The study was carried out in the central patio of two educational units.

In another work, the authors created an air-quality measurement system taking into account parameters such as CO_2_, No_2_, PM (particle matter) levels, temperature, and humidity [15]. This was achieved using sensors and a communications module. The used sensors were inexpensive and required extensive calibration given the interactions of temperature and humidity. In [20], low-cost air-quality sensors were used, such as those for measuring the level of particles, SO_2_, NO_2_, CO, O_3_, and CO_2_; in this system, the sensors send the data directly to an application through the LoRaWAN network, and the collected data are presented through an interface that allows to see the evolution of the measured parameters through graphs with a granularity of day, week, or month. The results of the work confirmed that LoRaWAN is appropriate for this type of monitoring solution. On the other hand, in [22], an IoT system was proposed for monitoring environmental parameters such as temperature, air pressure, humidity, noise, and air quality; the collected data are sent to a cloud server that saves and displays the measurement data. On the other hand, in [25], two stages of an air-quality monitoring system were established to facilitate the environmental changes in the regional air condition and early warning system. First, the air-quality sensors from Arduino and LoRa were integrated; the sensor nodes were then deployed on a university campus. Another work is the one presented in [2]; in this work, the authors proposed a monitoring system for moisture in the wood of buildings with cultural significance. The sensing solution was composed of a LoRaWAN communication device and a cloud application that allows remote visualization and control of the building’s nodes. The results showed that the proposed system can be applied inside buildings, despite obstacles such as thick walls and complex architectural structures. In [23], an environmental monitoring solution with parameters such as temperature, humidity, barometric pressure, and ambient light was proposed. The nodes were attached to a vehicle that shows the behavior in motion. The gateway was connected to the servers which deliver the gathered data to the end users.

In [16], WaterGrid-Sense is used, which is an intelligent interface platform; this platform has an integrated LoRa module that uses class A LoRaWAN protocol and has two sensor slots: one for a pressure sensor and the other for a pulse sensor. This intelligent platform was used for the management of a real-time intelligent water system, which was implemented at the Council for Scientific and Industrial Research (CSIR) campus. Another work is [21], in which a device was created to help monitor water quality in real time and identify abnormalities through a phone application. The device was created with the purpose of helping fish farmers; the device has different water sensors such as pH, water level, temperature, dissolved oxygen, total dissolved solids, oxidation, and turbidity sensors. The anomalies detected by the sensors activate different actuators such as the aerator, water filter, peristaltic pump, water pump, fish feeder, and heater to solve the problem. On the other hand, in [4], a comparison of LoRaWAN, Sig-Fox, and NB-IoT technologies is made; for this, a smart water grid is simulated using the NS-3 simulator. In this work, several problems in the water infrastructure were detected through the use of sensors; an example of these problems was the pipe leaks. With the results of the simulation, the conclusion was reached that NB-IoT has better scalability compared to the other two technologies.

#### 3.1.3. Mobility

Mobility is another area that benefits from LoRaWAN technology. Applications range from motion-sensor data communication to driving assistance using the movement detection of animals and people.

In [26], a project was developed in which it was planned to monitor garbage trucks in real time. To monitor them, four devices were used: two commercial and two assembled ones.

In [27], a LoRaWAN-based tracking system was implemented for the vehicle fleet of the municipality of Kozani, Greece. An experimental study was carried out with the objective of comparing the LoRaWAN network developed using two GPS trackers: an industrial solution and a customer solution. For this, a comparison is made between two types of sensors, RAK7200 and TTGO-TBeam, in terms of the number of packets and signal strength. The result shows the benefits of using external antennas; the most effective was the RAK7200 sensor with a 3dBi external antenna.

In [28], a service based on the use of parking sensors buried in the asphalt is presented to be able to detect if there is a vehicle at a certain distance above the sensor. The sensors were placed in two different geographical areas. In the first zone, 45 sensors were placed at a range of 250 and 385 m, while in the second, 24 sensors were placed at a distance between 25 and 60 m. The first scenario showed higher packet losses due to the distance from the gateways and the different obstacles presented in the way.

In [29], a road-condition monitoring system was proposed. For this, mobile sensors connected to a LoRaWAN network were used. The accelerometer and GPS of a smartphone were also used to measure vibrations in order to know the road conditions. The results allowed obtaining the classification of potholes, speed breaks, and damaged or patched streets. In [30], a solution for detecting and recognizing animal or people movement on the roadside to prevent accidents was proposed. This solution was focused on autonomous vehicles. LoRaWAN mesh technology was used to provide the data transmission of the remote networks and the main station, and the data were analyzed in a microcloud infrastructure.

#### 3.1.4. Health

Health is another area in which LoRAWAN technology is frequently applied. In [31], the use of LoRaWAN is proposed to monitor the health of workers and maintain efficiency during their work activity, analyzing working environment factors such as temperature, light, CO_2_ concentration, pressure, etc. On the other hand, in [6], a theoretical model is proposed to create a wireless monitoring device, which sends electrocardiogram and other data by applying machine-to-machine communication. Further, in [32], the creation of a device that monitors physical activity in elderly people is proposed, integrating an IMU (inertial measurement unit) to the sensors. Also, in [17], the implementation of an alert system for mine workers is proposed, taking into account the data obtained from smoke sensors and using respiratory and cardiac data. The study presented in [33] deals with a solution based on IoT devices and the deployment of several LoRaWAN gateways to geolocate vulnerable people, in which the location can be obtained at a range of 40 to 60 m.

### 3.2. Devices Used in LoRaWAN Applications

LoRaWAN applications use various hardware devices. Table 7 shows a summary of these devices. The table is structured as follows: the first column indicates the reference of the work; the second is the column of sensors, antennas, and gateways; and the third is the column corresponding to the boards and modules used in solutions. The denomination “N/A” (Not Available) refers to those works where the type of device related to the field is not mentioned.

Additionally, some elements that were used in various papers like software, libraries, and others can be seen in Table 8.

As can be seen in the table, the most used boards and modules are Arduino, followed by Raspberry Pi and devices manufactured by Semtech and STM Microelectronics. In general, the selection of these market-leading devices is due to their features of functionality, affordability, and extensive support, making them attractive options for a variety of projects and applications. Regarding the sensors, there is a great variety corresponding to the objectives of different applications mentioned in Table 6.

### 3.3. Other Works Analyzing Different Aspects of LoRaWAN

This section explains the previous works where the different characteristics of LoRaWAN technology are analyzed, performance studies of LoRaWAN are executed, and the comparative studies where different LPWAN technologies are contrasted.

First, we discuss the previous works where different characteristics of LoRaWAN technology are analyzed.

In [8], a review of the literature is carried out indicating various applications of LoRaWAN technology, focused mainly on intelligent parking systems. The sensors in these studies were divided into seven different types including camera, ultrasonic, cellular, infrared, radar, magnetometer, and others. Regarding the used wireless protocols, LoRa represented 5% of the total solutions, while other protocols such as ZigBee and IEE 802.15.4 represented 60% and 25%, respectively. The authors also indicate that security implementations have not been taken into account, generating vulnerabilities at the time of data collection.

In a similar way, in [38], the areas where LPWAN technologies can be applied are discussed, such as smart cities, water monitoring, environmental monitoring, energy monitoring, emergency services, logistics and automobiles, manufacturing, agriculture, and health, among others. The study also talks about characteristics such as coverage (rural vs. urban, outdoor vs. indoor), capacity (one-way, two-way, scalable), cost of the equipment, cost of the network and its scalability, the use power (efficiency, battery lifetime, latency), and features such as deployment scenarios or specific requirements. Additionally, the authors analyze the different protocols used in the solutions and suggest some best-practice considerations for future advantages, e.g., a reduction in complexity in terms of hardware.

Additionally, in [50], the authors perform an empirical study of different IoT applications, ranging from emerging applications such as smart cities, factory automation, and Industry 4.0 to traditional applications such as agriculture, transportation, and smart homes. LoRaWAN is mentioned as a long-range communication standard with a coverage of 5 km in urban areas and up to 20 km in rural areas.

Moreover, in [51], the authors present different applications of wireless sensors such as military, agriculture, soil monitoring, and water quality, among others. The authors propose a hybrid architecture that would increase the reliability and precision of communications. Additionally, the authors conclude that LoRaWAN is a promising technology for future IoT applications.

In addition to this, in [58], an introduction to LoRa and LoRaWAN is presented where the different technical aspects of those technologies are studied, such as modulation and spread factor. The author also analyzes the different types of applications that use LoRa and LoRaWAN technologies, such as environmental monitoring, livestock tracking, fall detection, fire detection, home security, water monitoring, smart parking lots, smart irrigation, etc.

Finally, in [60], projects with the potential to improve the quality of life of citizens by implementing the concept of smart cities are analyzed. Some of these projects were related to the remote monitoring of health, waste management, and public lighting using LEDs. The authors also highlight that the frequencies used by LoRaWAN can be used almost anywhere, and the experiments carried out showed that there could be a real coverage of 2.39 km in urban areas. Therefore, it is suggested to solve or mitigate local problems such as smart parking, counting the number of vehicles, or public lighting.

Next, we discuss the performance studies of LoRaWAN.

In [37], the advantages, disadvantages, and risks of adopting IoT devices are analyzed in two case studies. The case studies demonstrated that some of the organizational benefits were a reduction in costs, improvement in the speed and efficiency of the services by having information in real time, improvement in the organization’s reputation, and the flexibility of the services. On the other hand, some of the found organizational risks were conflicts with data privacy, difficulty of interoperability and integration, and data security breaches. These risks and benefits are studied in relation to organizational conditions and consequences.

In a similar way, in [62], the design of a LoRaWAN module together with a gateway is proposed, to facilitate data transmissions in a smart-city scenario. The usage mechanism consisted of four main steps. First, the RN2483 module was configured, and the communication messages were generated using Arduino Mega; second, the message from the module was transmitted to the gateway; third, the transmission of the message from the gateway to the server was executed; and fourth, the monitoring of the transmitted messages and the quality of the signal was performed. The proposed technology enabled reliable communications at a range of up to 6 km. The study was carried out using a 125 kHz bandwidth and a spreading factor (SF) of 7. The received signal strength indicator (RSSI) level ranged from 64 dBm to 111 dBm, while the theoretical value for SF7 was 123 dBm.

Moreover, in [64], the authors talk about an experiment performed on a campus to measure connectivity and try to answer the question if LoRaWAN technology works well in a real environment. This experiment measured the RSSI (received signal strength indicator), SNR (signal-to-noise Ratio), and PDR (packet-delivery ratio) to assess the data delivery performance of LoRaWAN technology. The results showed that the RSSI decreases depending on the communication distance, while SNR remains stable with distance and PDR fluctuates significantly.

Finally, we discuss the comparative studies where different LPWAN technologies are contrasted.

In [35], a comparison of five technologies, i.e., NB-IoT, SigFox, Telensa, Ingenu, LoRa, is made. By comparing these five technologies, it is concluded that LoRa and SigFox have the longest device lifetime, best data adaptive rate, best network capacity, and lowest costs compared to the other technologies. On the other hand, this work also concludes that NB-IoT is the one with the best quality of service and the lowest latency time. On the other hand, in [40], three classes of devices of LoRaWAN are described, i.e., A, B, and C; subsequently, a study of the performance of class B devices is carried out compared to class A devices. The authors conclude that the class B devices have a lower delay in downlink traffic compared to class A devices, and class B devices have a lower packet loss rate of downstream traffic.

Furthermore, in [41], a framework is used to compare the price of LPWAN deployment in urban and rural areas; in this work, the main cost factors for the deployment of the LPWAN network are identified, such as the cost of equipment, installation cost, spectrum cost, and electric power cost. Additionally, with NS-3 simulations of LoRaWAN and SigFox technologies, it was concluded that the impact of inter-technology interference on scalability in the unlicensed band is negligible under low traffic conditions.

Further, in [42], simulations are carried out and the performance of LPWAN technologies is evaluated in terms of signal propagation, coverage, and energy conservation; in LoRaWAN, if the sensitivity of the end nodes is increased by 10%, they can increase coverage by 142% without repercussions on energy consumption. In addition, the use of IoT devices in environments with a temperature of −20 °C in LoRaWAN networks reduces their life by 53%.

It is worth mentioning, in [46], a comparison between NB-IoT and LoRaWAN is made. This article compares these technologies in terms of the quality of service, cost, and energy consumption. In this work, the authors conclude that LoRaWAN is the one with the lowest costs and energy consumption, while NB-IoT has a better quality of service. Subsequently, experiments are made in different environments such as underground, underwater, and through metal in order to know the percentage of loss of a transmitted signal. After the experiments, it was concluded that NB-IoT has less signal loss in the different environments; however, it is the most expensive and consumes a greater amount of power compared to LoRaWAN.

In addition, in [1], a study is carried out in which LoRaWAN, SigFox, and NB-IoT are compared in terms of quality of service, payload length, reach, latency, and power consumption. This paper concludes that Sigfox and LoRaWAN provide advantages in terms of battery life, scalability, payload length, and cost, while NB-IoT has better quality of service, longer payload length, and lower latency.

Moreover, in [47], the authors carried out several simulations in OMNeT++, discovering that intense LoRaWAN networks can operate with variable traffic and high speed but will consume a large amount of energy and have a large number of collisions.

#### 3.3.1. Geolocation

Geolocation is a very useful tool that has been used in recent years, from delivery services to social networks. In this area, LoRaWAN devices have also been used to monitor the health of a person in a given location, as well as for livestock tracking applications. For example, ref. [43] describes the implementation of a livestock monitoring system using GPS, temperature, and humidity sensors.

#### 3.3.2. Security

One of the crucial aspects within the LoRaWAN implementation is security. Faced with this situation, many authors have carried out different studies on the security of LoRaWAN.

Several of the articles theoretically carry out the analysis of vulnerabilities present in LoRaWAN devices. In [53], the different risks that IoT devices have in different areas such as smart cities, home automation, and agriculture, among others, are discussed. The different threats that exist in the different layers are also analyzed, e.g., sensor layer and network layer, among others. Four different technologies, i.e., blockchain, fog computing, edge computing, and machine learning, are proposed to increase the level of security in IoT devices. On the other hand, in [54], the vulnerabilities present in various network technologies such as LoRaWAN, ASV, and ZigBee are analyzed. Most of the attacks consisted of replay attacks, eavesdropping, and bit-flipping attacks.

Additionally, in [48], the privacy implications of a drop in signal strength when a vehicle obstructs the sensor devices of a LoRaWAN-based smart parking lot are exploited. In this work, it is indicated that through an offside-channel attack, the attacker can collect data passively from great distances. Based on the use of supervised machine learning techniques based on neural networks, the attacker can estimate parking occupancy with an accuracy of up to 97%, while the random forest approach achieves an accuracy of more than 98%.

#### 3.3.3. Performance Improvement

Node lifetime extension is very important in LoRaWAN, and various methods can be used to achieve this end. For example, one way to improve performance is by improving supplies as well as using sleep mode to lower power consumption. In this area of study, there are several works that have been executed.

In [36], the LTC3588 energy harvesting module was used together with a solar panel and a supercapacitor to provide power to a microcontroller and a wireless sensor without the need for them to be connected to a battery; with this, it was achieved that the devices can have a sustainable operation. In [39], a class A end node was used to apply two methods for extending its working life period: the first was energy harvesting (EH) using a solar panel, and the second was the use of a deep sleep method. In [44], simulations of networks with backscatter communication were carried out, which had Backcom devices; these devices collected energy and transmitted data without the need for a battery, thus prolonging their lifetime. However, these devices generated a high rate of collisions when transmitting data. On the other hand, in [45], an algorithm called APRA was used, which varies the transmission priorities and signal processing dynamically to maximize the quality of service, avoid signal collisions, and improve the useful lifetime of the batteries. Also, in [34], the Wake-Up Radios technique was used, so that with the help of an automaton, the main node changes dynamically; it is important to indicate that the main node is chosen depending on the frequency with which it sends or receives signals, causing the rest of the nodes to enter a standby state, thus improving battery life.

In addition, there are works that with an improvement in the methods of sending information seek to increase the data-collection ratio without increasing energy consumption and thus improve the level of communication and scalability of LoRaWAN networks. Among these works, we can mention the following.

In [49], the study of a LoRaWAN network with different sensors is carried out, which generates asymmetric network traffic. In this work, it is demonstrated that the usage of Slotted ALOHA in the MAC layer helps to improve network performance; the simulations for this demonstration were carried out with asymmetric traffic of small, medium, and large volume.

Additionally, in [56], a simulation is carried out to create a network of wireless sensors that have a higher data collection rate than that established in the LoRaWAN standard and at the same time have an efficient use of energy. To achieve this, in this paper, the authors use a recursive linear prediction algorithm on the server side and a prediction error control algorithm on the end-device side. On the other hand, in another work, a modification of the LoRaWAN communication protocol is presented in which the payload data is encoded in custom frames to improve data transmission speeds [57].

On the other hand, in [59], the combination of LoRaWAN, SigFox, and GSM in a hybrid device is proposed; the information collected by the communication technologies is sent to a cloud platform and synchronized by an open platform communications (OPC) server to increase the reliability of the collected data.

In [63], the scalability and reliability of LoRaWAN in long-range communications are evaluated through a simulation model carried out in an NS-3 network simulator, with the result that multiple gateways considerably improve the reliability of the network. In addition, in [52], the performance of different adaptive data rate (ADR) schemes is evaluated. For this, in this work, several schemes are used in order to optimize the sending of data, for example, the use of a machine learning model to predict the metric that is being monitored.

In [55], an approach is proposed that allows modeling and calculating the behavior of the LoRaWAN network taking into account the availability of independent radio frequency channels. Because this scheme depends on the intensity of the channel traffic, this work endeavored to find an optimal traffic distribution using dynamic programming. Another interesting work is [61], where a large number of smart meters are used to manage an electrical network in real time; device management in the LoRaWAN network was optimized through the use of frequency and time multiplexing, thus allowing 98.45% of the packets sent to be received.

## 4. Discussion

**Figure 1:**
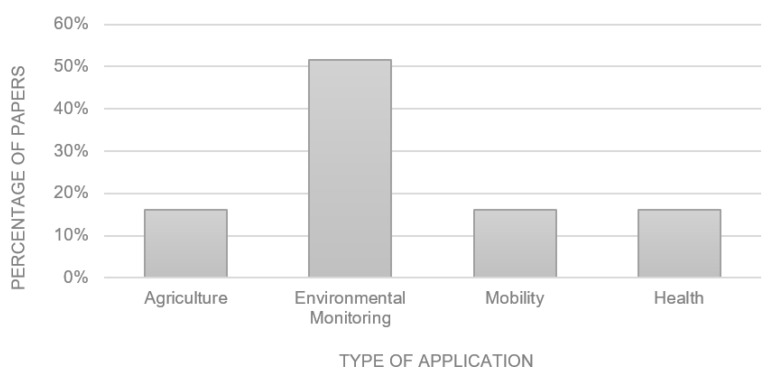
Categories of the solutions of the analyzed previous works.

The previous works reviewed in this paper develop different applications of different categories. In this aspect, the largest percentage of the articles carry out work on monitoring the environment, for example, monitoring temperature, humidity, etc. The second largest percentage of previous works are dedicated to developing or explaining solutions from the agriculture, mobility, and health categories. What has been described above can be seen illustratively in Figure 1.

**Figure 2:**
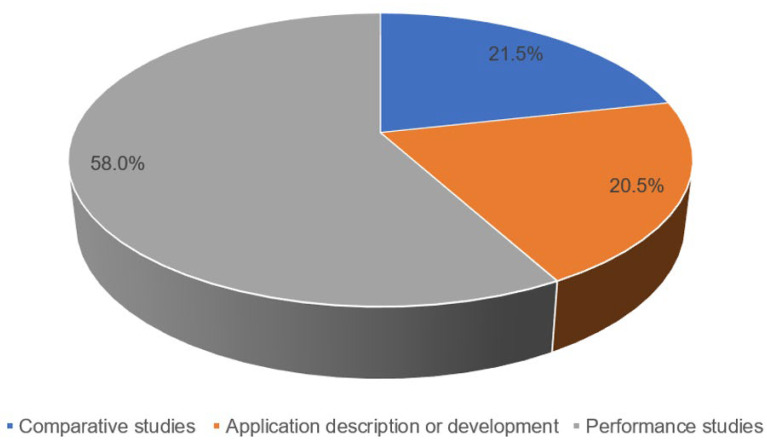
Distribution of the categories of “Other works”.

The “Other works” category was composed of papers from three subcategories. The first subcategory refers to works of comparative studies; these works compare the different LPWAN technologies, as well as the different algorithms they use. Comparisons often encompass features such as range, latency, deployment costs, and device lifetimes. The second subcategory of previous works dedicates their content in general applications; these works describe the different applications that can be used in different scenarios using LoRaWAN technology. Finally, the last subcategory is made up of works that are dedicated to carrying out studies to verify if some prototypes of LoRaWAN devices comply with the performance of the market standards. The distribution of these subcategories can be seen in Figure 2.

**Figure 3:**
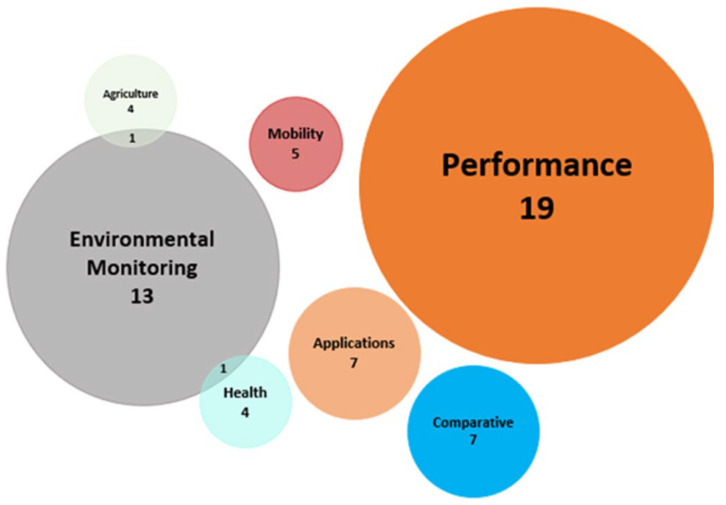
LoRaWAN application categories and their respective numbers of previous works.

In addition, in Figure 3, you can see the different LoRaWAN application categories and their respective number of previous works. Likewise, it is observed that not all articles belong to a single category but rather there are some that belong to two categories.

**Figure 4:**
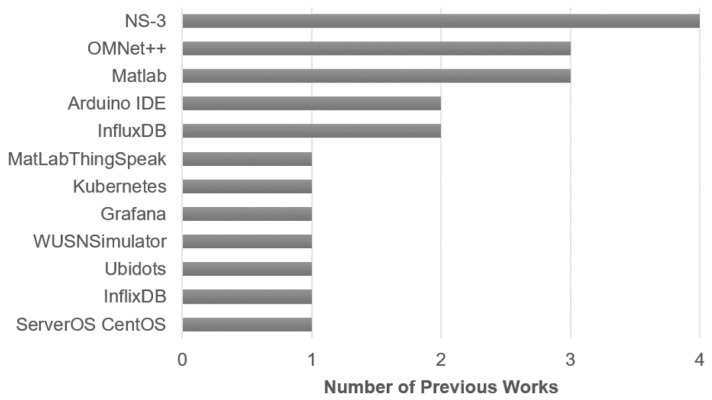
Frequency of use of software tools.

It is also important to indicate that in the previous works, different types of devices and tools are used, which can be categorized into (a) software, (b) boards and modules, and (c) sensors, gateways, and antennas. In Figure 4, it can be seen that the most used software is NS-3, which is used to perform LoRaWAN simulations; secondly, Matlab is used, which is also used to perform LoRaWAN simulations and additionally for data processing. After these two tools, three software tools are used most frequently: (a) OMNeT++, which is also a simulator, (b) InfluxDB, which is a database, and (c) Arduino IDE, which is a software used to program the boards.

**Figure 5:**
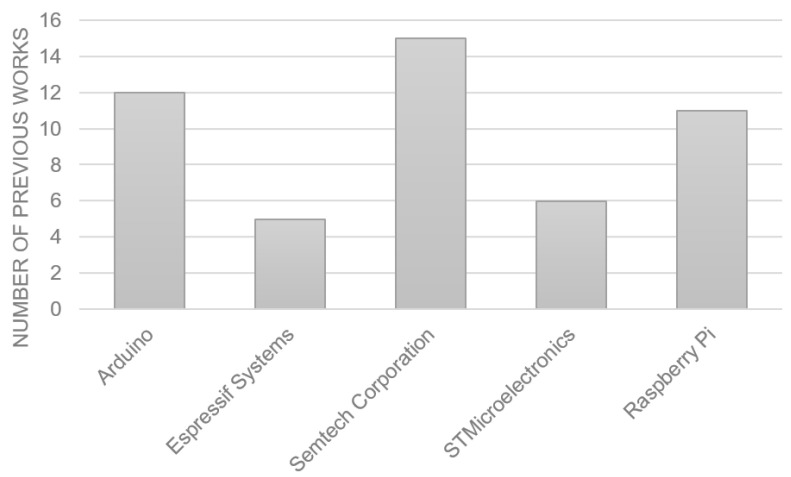
Most used board and module types and brands.

Additionally, in Figure 5, you can see the brands of boards and modules that are used mostly in different projects. On the horizontal axis, you can see the brand of the different devices, and on the vertical axis, the number of previous works that use these devices.

## 5. Conclusions

The number of articles that have been placed in the “Other works” category is equivalent to 52% of the analyzed papers, which indicates that LoRaWAN is a relatively new technology for which many applications are still expected to be carried out in various real environments. In this sense, measuring the performance of this technology and generating proposals for improvement is essential for its widespread adoption in real applications. Based on this background, in Figure 2, it can be seen that 21% of the papers correspond to improvements in LoRaWAN technology in terms of energy consumption and data transmission, which indicates an interest of the scientific and technological community in the improvement of this technology.

The classification of the papers in the different practical applications presented in Figure 4 indicates that this technology can be used in more than one application that includes areas such as health, agriculture, or security. In this aspect, although the use of LoRaWAN technology is still limited compared to other traditional wireless communication technologies, its use could be widely extended with the extension of the field of IoT and smart cities.

In conclusion, it can be inferred that the research questions have been successfully addressed, with the exception of the primary areas of study within the realm of LoRaWAN. This exception arises due to the fact that only 48% of the papers analyzed correspond to LoRaWAN applications.

Additionally, it can be concluded that the most used brand for boards and modules in real applications is Semtech Corporation with 15 projects, closely followed by Arduino and Raspberry Pi with 12 and 11 projects, respectively, positioning them as market leaders in the LoRaWAN applications that were studied.

The improvements that have been implemented in LoRaWAN correspond to security, energy performance, and packet delivery; additionally, there is a trend toward improvements in energy performance.

In this literature review, it has been observed that LoRaWAN is a promising technology that enables low-energy-cost communications. In addition, thanks to spread spectrum modulation, there is wide coverage and greater signal penetration through buildings and other obstacles. By working on unlicensed frequencies, the cost of contracting is avoided, making it an interesting option for many applications. For all this, LoRaWAN is a low-cost, long-range, and low-economic- and energy-cost technology, ideal for applications that require low data rates, such as environmental sensors, smart meters, asset tracking, and remote monitoring.

## Figures and Tables

**Table 1:** Search strings used in the first query.

Database	Search String	Number of Articles
IEEE Xplore	Lorawan AND Sensors AND Applications	324
Springer Link	Lorawan AND Sensors AND Applications	1474
SCOPUS	Lorawan AND Sensors AND Applications	445
ACM	Lorawan AND Sensors AND Applications	345

**Table 2:** Search strings used in the second query.

Database	Search String	Number of Articles
IEEE Xplore	(((“Document Title”: LoraWAN) AND (“Document Title”: sensors) AND (“Document Title”: applications)) OR ((“Abstract”: LoraWAN) AND (“Abstract”: sensors) AND (“Abstract”: applications)))	101
Springer Link	((All: loraWAN) AND (All: sensors) AND (All: applications)) OR ((Title: loraWAN) AND (Title: IoT) AND (Title: “Internet Of Things”)) AND (Publication Date: 1 January 2020 TO 31 December 2023)	57
SCOPUS	TITLE-ABS (lorawan AND sensors AND applications) AND (LIMIT-TO (LANGUAGE, “English”)) AND (LIMIT-TO (PUBYEAR, 2023) OR LIMIT-TO (PUBYEAR, 2022) OR LIMIT-TO (PUBYEAR, 2021) OR LIMIT-TO (PUBYEAR, 2020))	239
ACM	[All: lorawan] AND [All: sensors] AND [All: applications] AND [[Keywords: lorawan] OR [Keywords: iot] OR [Keywords: “internet of things”]] AND [E-Publication Date: (1 January 2020 TO 31 December 2023)]	109

**Table 3:** Search strings used in the third query.

Database	Search String	Number of Articles
IEEE Xplore	(((“Document Title”: LoraWAN) AND (“Document Title”: sensors) AND (“Document Title”: applications)) OR ((“Abstract”: LoraWAN) AND (“Abstract”: sensors) AND (“Abstract”: applications)) AND (“Author Keywords”: LoraWAN) AND ((“Author Keywords”: IoT) OR (“Author Keywords”: Internet of Things)))	30
Springer Link	((All: loraWAN) AND (All: sensors) AND (All: applications)) OR ((Title: loraWAN) AND (Title: IoT) AND (Title: “Internet Of Things”)) AND (Publication Date: 1 January 2020 TO 31 December 2023)	19
SCOPUS	TITLE-ABS (lorawan AND sensors AND applications) AND (LIMIT-TO (LANGUAGE, “English”)) AND (LIMIT-TO (PUBYEAR, 2023) OR LIMIT-TO (PUBYEAR, 2022) OR LIMIT-TO (PUBYEAR, 2021) OR LIMIT-TO (PUBYEAR, 2020)) AND (LIMIT-TO (EXACTKEYWORD, “Internet Of Things”) OR LIMIT-TO (EXACTKEYWORD, “LoRaWAN”))	173
ACM	[All: lorawan] AND [All: sensors] AND [All: applications] AND [Keywords: lorawan] AND [[Keywords: iot] OR [Keywords: “internet of things”]] AND [E-Publication Date: (1 January 2020 TO 31 December 2023)]	14

**Table 4:** Search strings used in the fourth query.

Database	Search String	Number of Articles
SCOPUS	TITLE-ABS (lorawan AND sensors AND applications AND monitoring) AND (LIMIT-TO (PUBYEAR, 2023) OR LIMIT-TO (PUBYEAR, 2022) OR LIMIT-TO (PUBYEAR, 2021) OR LIMIT-TO (PUBYEAR, 2020)) AND (LIMIT-TO (LANGUAGE, “English”)) AND (LIMIT-TO (EXACTKEYWORD, “LoRaWAN”)) AND (LIMIT-TO (EXACTKEYWORD, “Internet Of Things”) OR LIMIT-TO (EXACTKEYWORD, “IoT”))	23

**Table 5:** Number of articles: partial review vs. complete review.

Database	Search String	Number of Articles
IEEE Xplore	30	23
Springer Link	19	17
SCOPUS	23	18
ACM	14	3
Mendeley	0	2

**Table 6:** References organized by their application areas.

Area	References
Agriculture	[9,10,11,12,13]
Environmental Monitoring	[2,4,10,14,15,16,17,18,19,20,21,22,23,24,25,26]
Mobility	[27,28,29,30,31]
Health	[6,17,32,33,34]
Other areas	[1,8,35,36,37,38,39,40,41,42,43,44,45,46,47,48,49,50,51,52,53,54,55,56,57,58,59,60,61,62,63,64,65]

**Table 7:** Sensors, antennas, gateways, boards, and modules used in the analyzed solutions.

Ref	Sensors, Antennas and Gateways	Boards and Modules
[8]	Wide-angle camera, cell phone sensors, ultrasonic sensors, LIDAR, geomagnetic sensors, radar sensors, TSOP1738	Arduino Uno
[14]	UVM-30A	ESP32
[36]	BMP180	ASR6501, LTC3588, RAK831
[26]	Sony-Semicon CXD5603GF, Ublox Max 7Q, Ublox Neo-6M, 6 dBi omnidirectional antenna, RAK7200	STM32L073, STM32L1 MCU, SX1276, SX1272, Arduino Uno, Raspberry PI 2, TIGO-T-Bean, RAK5205 WisTrio
[39]	iM880B	N/A
[15]	Alphasense CO-B4, GP2Y1010AU0F, NO2-B43F	ESP8266, CMWX1ZZABZ-078
[43]	DHT11	SX1278, Raspberry Pi3, ESP8266
[27]	RAK7200, CXD5603HF	TIGO T-Beam, LORIX One, STM32L073, SX1276
[16]	Pressure sensor, pulse sensor, LoRa gateway	N/A
[44]	N/A	LoRa Backscatter
[31]	Temperature sensor, humidity sensor, pressure sensor, brightness sensor, CO2 sensor, oxygen sensor, Cisco Wireless Gateway	N/A
[46]	N/A	RAK831, Core-L073RZ, STM32L073, RFM95, SARA UBee, Raspberry PI 3B
[6]	Blood pressure sensor, glucometer, thermometer, ADAS1000-3	Arduino Mega, TI TMS320, SX1272
[32]	LPS22HB, BNO080, SPU0414HR5H-SB, TSOP7000	SMT324F69, CORTEX-M4, LORIX One, SX1276TP4056
[17]	MQ-8 gas sensor, horn, LED iode, heart rate sensor, breath sensor	SIM900 GPRS, Arduino Uno
[18]	GUVA-S12SD, analog sensor node	ESP32
[48]	N/A	Raspberry Pi, iC880A, RAK31, CXL 900-6LW
[2]	N/A	WiMOD iM881-XL, STM32L081
[29]	Accelerometer, GPS, gyroscope	N/A
[19]	ML8511, UVM30A, VEML6075	RFM95W, Arduino Mini Pro
[20]	PMA115S0-XXX	Raspberry PiHA, Arduino Mega, Dragino LoRa Shield, LoRaWAN HAT + antenna
[34]	Wake-Up Receiver, Wake-Up Radios	N/A
[9]	DHT-22	Raspberry Pi 3, Lora Shield, RFM95W, NXP OM13069, Arduino Uno
[21]	pH sensor, oxidation-reduction-potential sensor, water temperature sensor, water level sensor, turbidity sensor	N/A
[10]	N/A	ESP32, PIC, Raspberry Pi, SX1301
[22]	BME280	Sodaq Mbili, Arduino ATmega 1284p, RN2483, LM386, Raspberry Pi 3, RAK831
[23]	4 dBi antenna	Pycom FiPy Development Module, RHF0M301, Raspberry Pi
[53]	Ultrasonic sensors, camera sensors, smoke-detection sensors, temperature and humidity sensors	N/A
[30]	N/A	Módulos LoRa MESH
[56]	B57861	MiKroTik R11e-LRS, SX1301, Vega BS-1, STM32L151, SX1272
[57]	GPS6MV2, DHT11, MPU6050, BPM180	N/A
[58]	N/A	SX1272, SX1276, SX1278, Adafruit RFM96W, SX 1301, SX 1308, Dragino LG01-P
[33]	N/A	Fipy con PySensor Shield, PyTrack, SiPy Modules
[59]	Temperature sensor	LoRa module E32 Ebyte, Arduino Nano V3
[13]	N/A	Raspberry pi
[60]	DHT 22	RFM95W, Arduino Pro Mini Atmega328, RAK831
[62]	TTN-GW-868 gateway	RN2483, Arduino Mega
[64]	4.5 dBi antenna, 6 dBi antenna	Arduino Uno R3, SX 1272, SX 1301, MTAC-LoRa, Libelium LoRaWAN 900
[24]	Si1145 sensor	Raspberry Pi
[25]	Pms5003t sensor	Raspberry Pi 3, LoRa Shield

**Table 8:** Software, libraries, and others used in the analyzed solutions.

Ref	Software and Libraries	Others
[14]	MatLab ThingSpeak, Arduino IDE	N/A
[35]	LoRaSIM, NS-3, FLoRa, CupCarbon, PhySimulator	N/A
[36]	N/A	3.3 V 10 F supercapacitors, 10 V solar panel
[26]	LMIC Library, Tiny GPS ++	N/A
[39]	N/A	Panasonic BP 376634C
[27]	Server OS CentOS 8, InflixDB	N/A
[45]	LoRaSIM	N/A
[46]	Node-RED, Matlab, MySQL	N/A
[47]	FLoRa, OMNeT++	N/A
[32]	Influx DB	cell- LiPo, SPX3819, MCP4161
[18]	N/A	Powerbank
[29]	N/A	Logistic regression, ANN, SVM, convolutional neural network, support vector machine.
[20]	N/A	Plastic enclosure
[9]	Ubidots	Kalman filtering, decision trees
[10]	N/A	Powerbank
[22]	N/A	GL552B, solar panel
[12]	WUSN Simulator	Friis model, Peplinski model, complex refractive index model
[52]	NS-3, OMNeT++, FLoRa	N/A
[56]	Matlab	N/A
[59]	Influx DB, Gafana, Node-Red	N/A
[13]	Arduino IDE, LoraMAC-in-C software libraries	N/A
[61]	Matlab	N/A
[63]	NS-3, LoRaPhy, LoRaGWPhy	N/A
[24]	Raspbian OS, Python	N/A
[25]	Kubernetes, Docker, MySQL	N/A

## Data Availability

Not applicable.
